# Stereochemical course of hydrolytic reaction catalyzed by alpha-galactosidase from cold adaptable marine bacterium of genus *Pseudoalteromonas*

**DOI:** 10.3389/fchem.2014.00089

**Published:** 2014-10-13

**Authors:** Irina Y. Bakunina, Larissa A. Balabanova, Vasiliy A. Golotin, Lyubov V. Slepchenko, Vladimir V. Isakov, Valeriy A. Rasskazov

**Affiliations:** G.B. Elyakov Pacific Institute of Bioorganic Chemistry, Far Eastern Branch, Russian Academy of SciencesVladivostok, Russia

**Keywords:** Recombinant retaining α-galactosidase, marine bacterium, ^1^H NMR spectroscopy

## Abstract

The recombinant α-galactosidase of the marine bacterium (α-*Ps*Gal) was synthesized with the use of the plasmid 40Gal, consisting of plasmid pET-40b (+) (Novagen) and the gene corresponding to the open reading frame of the mature α-galactosidase of marine bacterium *Pseudoalteromonas* sp. KMM 701, transformed into the *Escherichia coli* Rosetta(DE3) cells. In order to understand the mechanism of action, the stereochemistry of hydrolysis of 4-nitrophenyl α-D-galactopyranoside (4-NPGP) by α-*Ps*Gal was measured by ^1^H NMR spectroscopy. The kinetics of formation of α- and β-anomer of galactose showed that α-anomer initially formed and accumulated, and then an appreciable amount of β-anomer appeared as a result of mutarotation. The data clearly show that the enzymatic hydrolysis of 4-NPGP proceeds with the retention of anomeric configuration, probably, due to a double displacement mechanism of reaction.

## Introduction

α-Galactosidase (α-D-galactoside galactohydrolase; EC 3.2.1.22) catalyzes the hydrolysis of terminal α-linked galactose residues in oligosaccharides and polymeric galactomannans. They have found a number of useful potential biotechnological and medical applications. An interesting application of α-galactosidase in biomedicine is the processing of B-red blood cells for conversion into universal O-red blood cells (Olsson et al., 2004). In medicine, it plays a crucial role in the treatment of Fabry's disease (Breunig et al., 2003) and for overcoming xenorejection for xenotransplantation (Ezzelarab and Cooper, 2005). α-Galactosidases are used in the food and feed industry to improve the quality of products containing oligosaccharides of raffinose series (Anisha and Prema, 2008).

α-Galactosidases are wide distributed in marine bacteria, especially among γ-proteobacteria (Ivanova et al., 1998) and *Bacteroidetes* (Bakunina et al., 2012), however, an α-galactosidase isolated from the marine bacterium *Pseudoalteromonas* sp. KMM 701 was the first biochemicaly characterized marine α-galactosidase. It catalyzes the hydrolysis of α-galactose residues from non-reducing end of B-trisaccharides and is capable for reducing a serological activity of B-red blood cells at neutral pH. Furthermore, the α-galactosidase is able to interrupt the adhesion of pathogens to human buccal epithelium (Balabanova et al., 2010). These properties showed the great therapeutic potential and opened up broad prospects for application of the enzyme in medicine. The α-galactosidase gene was isolated (GenBank DQ530422) and the amino acid sequence was reconstructed (UniProt Q19AX0). The marine bacterium *Pseudoalteromonas* sp. KMM 701 α-galactosidase has been found to belong to GH36 family in CAZy classification, according to its amino acids sequence (Balabanova et al., 2010).

The question of stereochemical outcome of enzymatic hydrolysis of glycosides is principal in the understanding of a catalytic mechanism and in classification of glycoside hydrolases. ^1^H NMR spectroscopy is a direct way for determining the stereochemical configuration of the product anomers.

The paper aims to produce the recombinant α-galactosidase of marine bacterium *Pseudoalteromonas* sp. KMM 701 (α-*Ps*Gal) and investigate stereochemistry of the glycoside hydrolysis for understanding the enzymatic mechanism of action.

## Materials and methods

### Rcombinant α-galactosidase construction

For the α-*Ps*Gal gene amplification, Encyclo Taq Polymerase (Eurogene), genomic DNA of the marine bacterium *Pseudoalteromonas* sp. KMM 701 and the gene-specific the upstream primer, G2-NcoI-for40: 5′-ATTACCATGGATGACGACGACAAGGCCGACACTAAATCATTTTATCGATTAGACA-3′, and the downstream primer, G3-SalI-rev40: 5′-ACACGTCGACTTACGCTTTGTTGAGCTCAAATATAAGC-3′ were used. The resultant PCR products were purified with agarose gel (Helicon). PCR was carried out in automatic amplifier “Eppendorf.” Restriction endonucleases and T4 DNA ligase were purchased from “Thermo Scientific.” The restricted PCR product and plasmid pET-40b (+) were purified from agarose gel with the use of a “Qiagen” column. The recombinant insert of the resultant plasmid 40Gal was sequenced using the automated PE/ABI 310 DNA sequencer and the PE/ABI-ABI PRISM BigDye Terminator cycle sequencing Ready Reaction Kit (PE Applied Biosystems). Preparation of *Escherichia coli* competent cells and heat shock transformation were carried out according to the standard methods (Sambrook et al., 1989).

For producing α-*Ps*Gal of marine bacterium *Pseudoalteromonas* sp. KMM 701 the recombinant plasmid 40Gal was transformed in to the *E. coli* Rosetta(DE3) cells. The obtained recombinant cells were grown on LB plate containing 25 μg/mL of kanamycin overnight at 37°C. A single colony was picked and grown at 200 rpt in 20 mL of LB, with 25 μg/mL of kanamycin at 37°C for 12 h. Overnight culture was transferred to 1 L of fresh LB with 25 μg/mL of kanamycin. When the cell density reached an OD_600_ of 0.6–0.8, 0.2 mM IPTG was added to induce the expression and the incubation was continued at 16°C up to 12 h at 200 rpt. The *E. coli* Rosetta(DE3) cells were transformed with the pET-40b (+) plasmid as a control.

### Recombinant α-galactosidase purification

All purification steps were carried out at +6°C. After harvesting, the transgenic *E. coli* Rosetta (DE3)/40Gal cells were resuspended in 200 mL of buffer A (0.01 M NaH_2_PO_4_, pH 7.7, 0.01% NaN_3_) and sonificated by ultrasonic treatment, then centrifuged at 10,000 g for 30 min. The supernatant was applied to a column (2.5 × 30 cm) of Macro-Prep (BioRad). Elution of the protein was performed with the use a linear gradient of NaCl concentration (0.05–0.3 M) in the buffer A. The enzymatic active fractions were collected and applied to a column (1 × 2.5 cm) of Ni-NTA agarose (Qiagen). Elution of the protein was carried out in buffer B (0.01 M NaH_2_PO_4_, pH 7.7, 0.01% NaN_3_, 0.04 M EDTA). The active fractions were collected and desalted with the use of Bio-Scale™ Mini Macro-Prep® High Q 1 mL cartridge, then incubated with enterokinase (Invitrogen) at 21°C for 15 h to cleave N-terminal plasmid overhang from the chimeric protein (32.5 kDa). Then, the protein solution is applied to a gel filtration column (1.5 × 170 cm) of Superdex 200 (Sigma) equilibrated with the buffer C (0.01 M NaH_2_PO_4_, pH 7.7, 0.01% NaN_3_, 0.1 M NaCl). All purification steps were examined by SDS-PAGE. The concentration of the protein was determined according to Bredford (1976). The obtained recombinant polypeptide is determined by the first 10 amino acids on an automatic sequencer Procise model 492 (Applied Biosystems, USA). All biochemicals and reagents were from “Thermo scientific” and “Sigma-Aldrich.”

### Enzyme essay

Enzyme activity of the α-*Ps*Gal was determined with 3.3 mM of 4-NPGP in 50 mM sodium phosphate buffer pH 7.5, at 20°C. One unit of the α-galactosidase activity was defined as amount of the enzyme that hydrolyzed 1 μmol of 4-NPGP per min. Total quantity of p-nitrophenol was determined spectrophotometrically at 400 nm (ε_400_ = 18300 M^−1^ cm^−1^).

### Molecular mass determination

The molecular size of the active α-*Ps*Gal after treatment of enterokinase was determined by gel filtration on the column of Superdex 200 (Sigma) (1.5 × 170 cm) in the buffer A at a flow of 0.16 mL/min at 6°C and calibrated using Bio-Rad standard molecular weight markers: Thyroglobulin bovine (670 kDa), γ-globulin bovine (158 kDa), Ovalbumin chicken (44 kDa), Mioglobin horse (17 kDa), Vitamin B_12_ (1.35 kDa). The molecular mass of the enzyme was determined by 12.5 % polyacrylamide gel electrophoresis (PAGE). The protein preparation was mixed with Laemmli sample buffer with heat treatment at 95°C, and then applied to PAGE. The SDS-PAGE gels were stained according to Laemmli (1970).

### ^1^H NMR spectra recording and analysis

Determination of the anomeric center configuration of the product of the hydrolysis reaction was monitored by ^1^H NMR spectroscopy. The experiment was performed at 20°C with the use of NMR DRX-500 spectrometer (Bruker, Germany). ^1^H NMR spectra were acquired over 32,000 data points using a spectral width 5000 Hz.

Prior to analysis by NMR, 0.6 mL 50 mM sodium phosphate buffer, pH 7.5, and substrate were dried on rotary evaporator and dissolved in 0.6 mL D_2_O. The deuterium-exchanged α-*Ps*Gal was obtained on vivaspin turbo 10 K MWCO (Sartorius, Germany). Chemical shifts were measured relative to an external standard—acetone (trace) in D_2_O. CH_3_—signal acetone was set to δ = 2.22 ppm. After recording of the original spectrum at τ = 0 min 6.0 mmol of the deuterium-exchanged p-nitrophenyl galactopyranoside in 0.6 mL of D_2_O, 0.1 mL of the deuterium-exchanged α-*Ps*Gal containing 1.14 U was added for initiation of the reaction. ^1^H NMR spectra were recorded automatically at intervals of 3 min for 60 min from the start of the reaction.

^1^H NMR anomer signals (αH-1) of α-4-NPGP, α-galactopyranose, and (β H-1) of β-galactopyranose, as well as proton signals of free 4-nitrophenol ring were analyzed and integrated by the standard software “TopSpin 3.2.” Integral intensities of signals of each anomer were calculated as a percentage of the total integral intensities of all signals of anomers and were plotted depend on time. The degree of substrate hydrolysis (4-NPGP) was defined as a percent ratio of the ^1^H NMR integral intensities of proton signals of free 4-nitrophenol ring at 8.109 ppm to proton signals of 4-NPGP at 8.275 ppm and also was plotted depend on time.

## Results

### Recombinant α-galactosidase production and characterization

**Figure 1:**
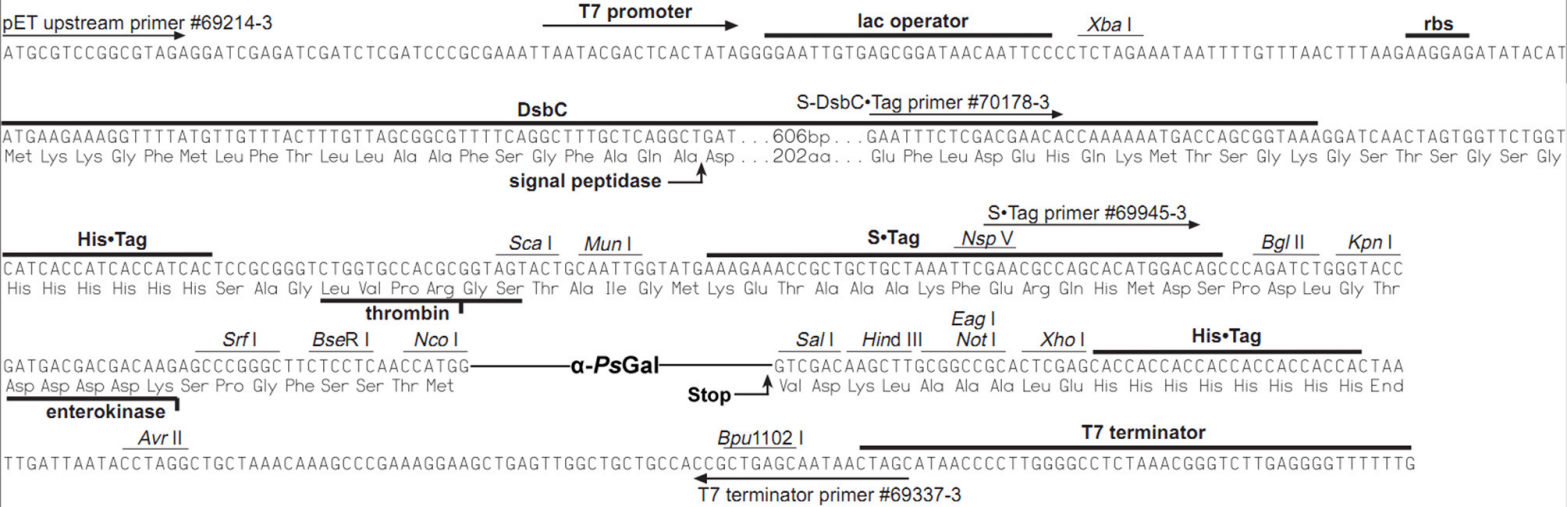
**The nucleotide and amino acid sequences of the α-*Ps*Gal expression region**.

The recombinant plasmid 40Gal of 8306 base pairs (bp) for the synthesis of the recombinant protein α-*Ps*Gal consisted of the NcoI/SalI-fragment of plasmid pET-40b (+) (Novagen) and the gene of 2130 bp corresponding to the open reading frame of the mature α-galactosidase of marine bacterium *Pseudoalteromonas* sp. KMM 701 (Figure 1).

The IPTG concentration of 0.2 mM, strain cultivation temperature of 16°C and duration of the cultivation of 12 h at 200 rpt were optimal conditions for the α-*Ps*Gal expression. The induced α-*Ps*Gal expression in 1 L culture of *E.coli* Rosetta(DE3)/40Gal cells yielded up to 10 mg of the functionally active protein with the specific activity 160 U/mg at the final purification step. α-*Ps*Gal has been found to provide the complete inactivation of serological activity of B-red blood cells and its conversion into the O-red blood cells in the same condition as for the native enzyme (data not shown).

**Figure 2:**
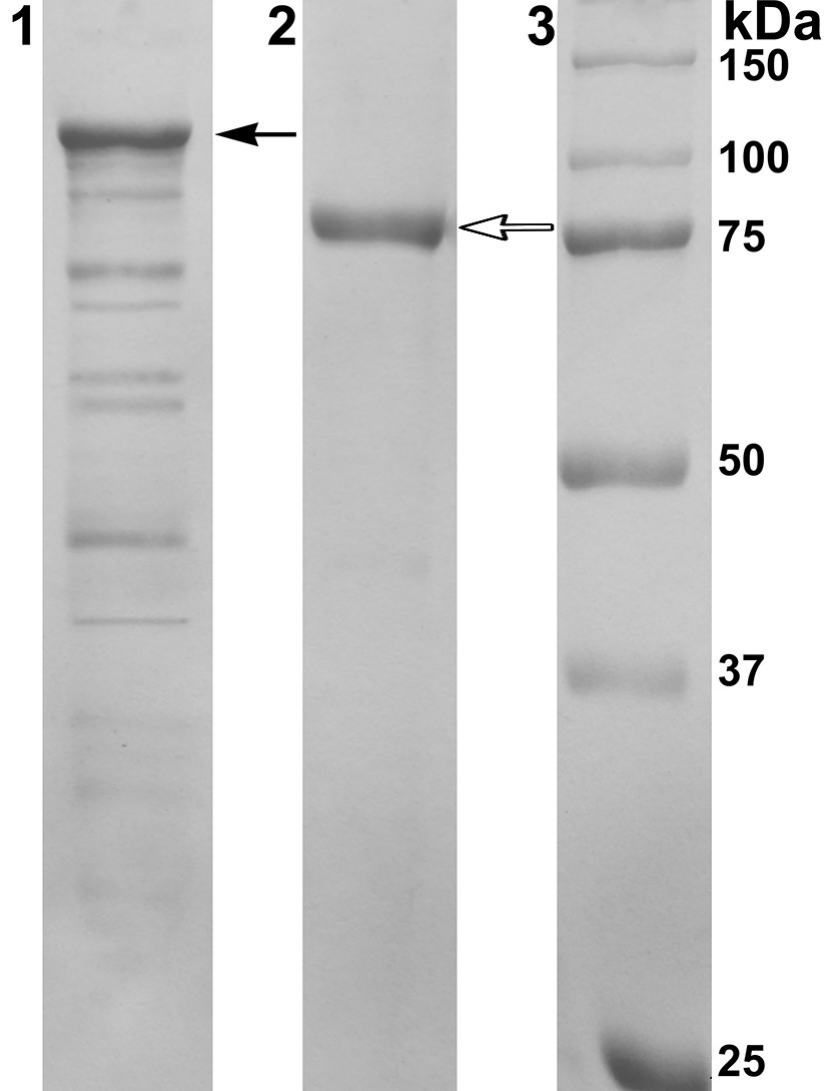
**Results of 12.5% SDS-PAGE electrophoresis**. Line 1: Cell extract of *E. coli* Rosetta(DE3)/40Gal; Line 2: α-*Ps*Gal after final purification step; Line 3: MW-standards. Filled arrow indicates the band corresponding to the chimeric protein with DsbC overhang (112.5 kDa); empty arrow indicates the band corresponding to the mature protein after enterokinase treatment (80 kDa).

PAGE-SDS showed a single band of the purified recombinant protein with a molecular mass of approximately 80 kDa corresponding to the one subunit of α-*Ps*Gal (Figure 2).

The molecular mass of the mature protein determined by gel filtration was to be 160 kDa, suggesting the two-subunit molecular organization of the active α-*Ps*Gal.

### ^1^H NMR spectra recording and analysis

**Figure 3:**
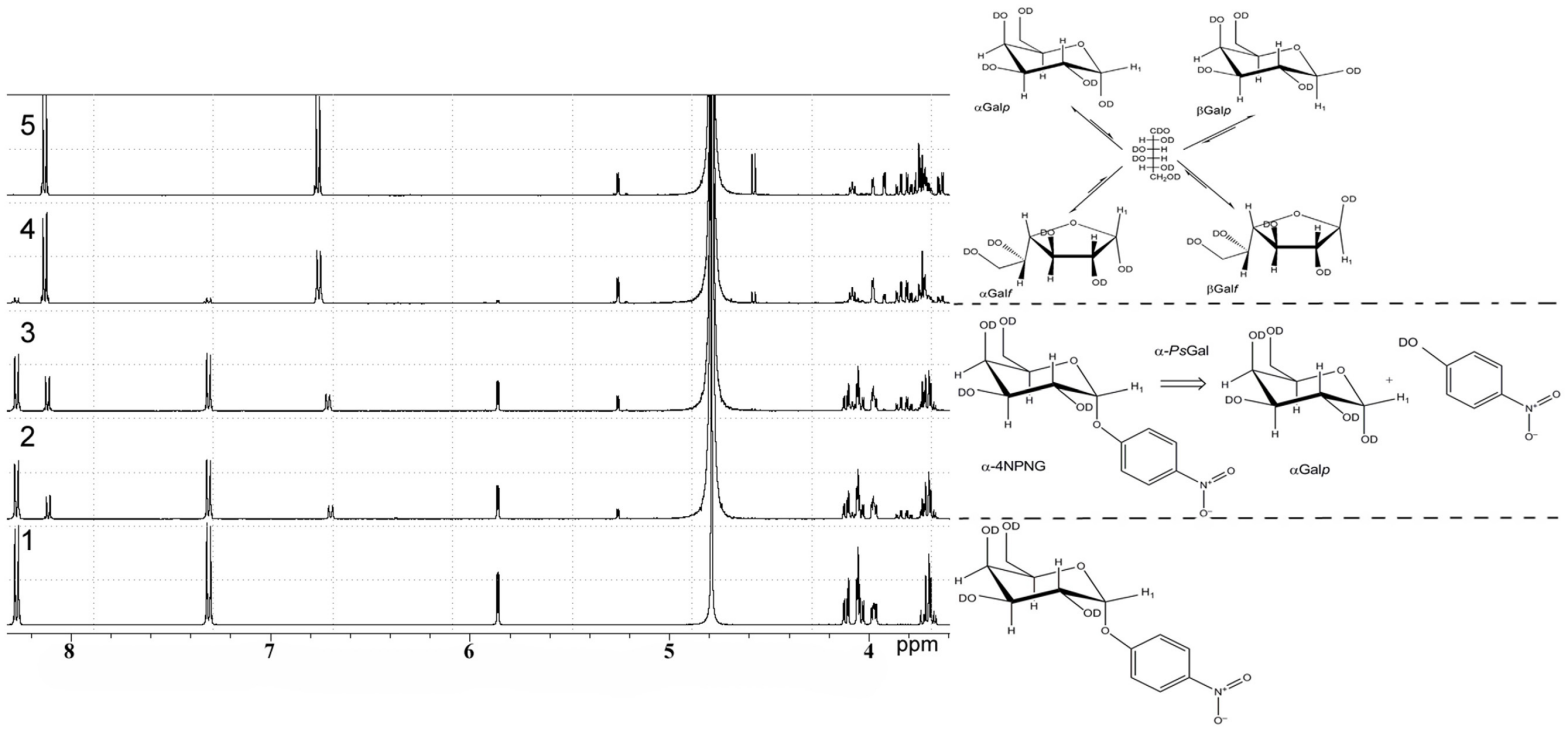
**^1^H NMR analysis of the stereo selectivity for the recombinant α-*Ps*Gal catalyzed reaction**. One-dimensional ^1^H NMR spectra of the deuterium-exchanged 4-NPGP prior to enzyme addition, at τ = 0 min (spectrum 1), at τ = 3 min (spectrum 2); at τ = 6 min (spectrum 3); at τ = 24 min (spectrum 4); at τ = 60 min (spectrum 5) after initiation of the reaction. (in 50 mM sodium phosphate buffer pH 7.5, at 20°C, D_2_O).

^1^H NMR spectra of deuterium-exchanged 4-NPGP and products of it's hydrolysis under the action of the recombinant α-*Ps*Gal are shown on Figure 3.

In ^1^H NMR spectrum 4-NPGP (Figure 3, spectrum 1, formula of the compound is shown on the right) one signal was a doublet at 5.84 ppm [*J*_(1, 2)_ = 3.43 Hz] and two groups signals at 8.28 and 7.31 ppm corresponded to αH-1 anomer and 4-nitrophenol ring protons in 4-NPGP, respectively. Moreover, signals in region of resonance from 4.13 to 3.96 ppm are observed. These signals corresponded to H-2, H-3, H-4, H-5, and H-6 protons of α-galactopyranose in 4-NPGP (Figure 3, spectrum 1).

After addition of the enzyme, the doublet signal was appeared at 5.26 ppm [*J*_(1, 2)_ = 3.8 Hz] during the first 3 min (Figure 3, spectrum 2, reaction equation is shown on the right). The doublet was identical in chemical shift to the αH-1 resonance of free α-D-galactopyranose (Angyal and Pickles, 1972). Signals arising at 8.11 and 6.68 ppm related to protons of free 4-nitrophenol formed through the reaction. Decrease in the amplitude of signals at 8.28 and 7.31 ppm indicates disappearance the substrate. A doublet signal at 4.57 ppm [*J*_(1, 2)_ = 7.8 Hz], which was identical in chemical shift to the H-1 resonance of free β-D-galactopyranose (β H-1) (Angyal and Pickles, 1972), appeared after 10 min of the reaction in accordance with the time of spontaneous mutarotation (Figure 3, spectrum 4). Signals of protons H-2, H-3, H-4, H-5, and H-6 of free α- and β-D-galactopyranose occurred at 3.40–4.20 ppm, minor signals at 5.21 ppm [J_(1, 2)_ = 3.30] rising at late time of reaction corresponded to β-D-galactofuranose (Angyal and Pickles, 1972) (Figure 3, spectra 4 and 5, equation of equilibrium of D-galactose is shown on the right Zhu et al., 2001). Signals in the anomeric region, which could indicate the emergence of new products of the reaction or formation of O-glycosidic bond in the spectra were not found.

**Figure 4:**
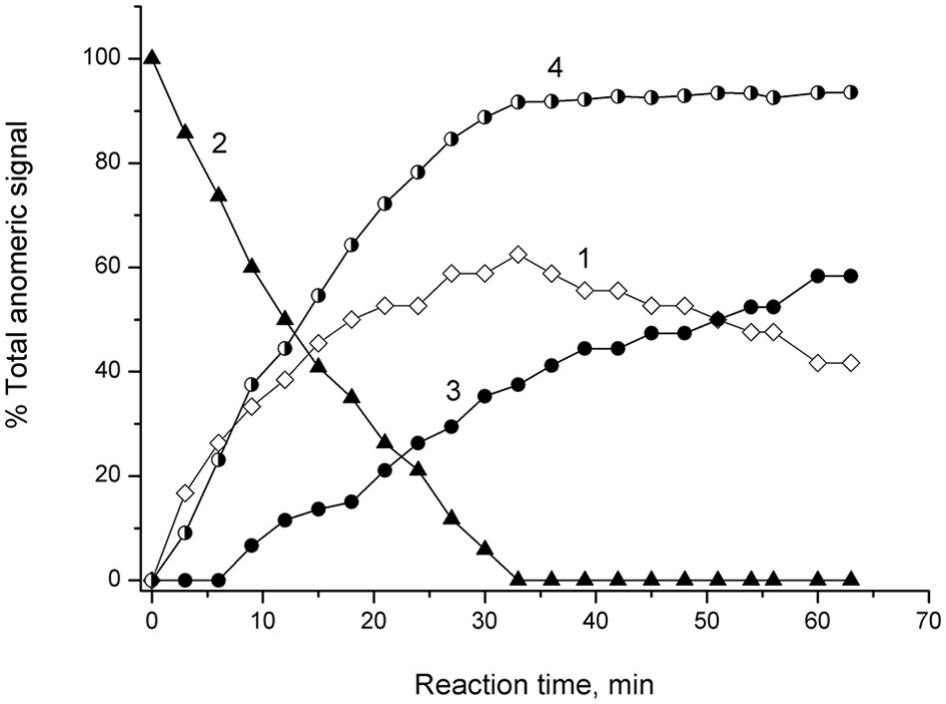
**^1^H NMR Monitoring of reaction of α-4-NPGP hydrolysis catalyzed by α-*Ps*Gal in 50 mM sodium phosphate buffer pH 7.5, at 20°C, D_2_O**. Experimental time-dependent changes in integral intensity of anomer signals. Curve 1: αH-1 of reaction product of galatopyranose at 5.26 ppm; Curve 2: αH-1 of 4-NPGP at 5.84 ppm; Curve 3: β H-1 mutarotation product of α-galatopyranose at 4.57 ppm; Curve 4: shows the change in the degree of hydrolysis of the substrate vs. time.

NMR spectroscopic monitoring of the hydrolysis of 4-NPGP by α-*Ps*Gal is shown on Figure 4.

Signal at 5.26 ppm the α-D-galactopyranose αH-1 appeared at the first minutes after the addition of α-*Ps*Gal (Figure 4, curve 1). Disappearance of doublet at 5.84 ppm of αH-1 of 4-NPGP (Figure 4, curve 2) simultaneously with appearance of the signal at 5.26 ppm of αH-1 of α-D-galactopyranose increasing in intensity (Figure 4, curve 1) were observed within next 30 min. On curve of the time-dependent changes in signal amplitude of β H-1 at 4.57 ppm lag-period were observed during 9 min (Figure 4, curve 3), when degree of the substrate hydrolysis reached up to ~30% (Figure 4, curve 4). After 33 min, the degree of substrate hydrolysis reached up to 98–100% (Figure 4, curve 4). After 60 min, the ratio of integral intensities of αH-1 and β H-1 signal was 40:60. Typical equilibrium ratio 31:69 between αH-1 and β H-1 D-galactopyranose anomers (Angyal and Pickles, 1972; Zhu et al., 2001) was formed for 180 min from the start of the reaction in experimental conditions pH 7.5 and 20°C.

## Discussion

Marine bacteria have been found to be a good source for α-galatosidases, which are stable at low temperatures and hence find use in medical and food industry applications. These enzymes appear to be also distinct from their terrestrial counterparts in substrate preference and catalytic efficiency. The first example was the enzyme from the marine bacterium *Pseudoalteromonas* sp. KMM 701 that displayed a good stability at low temperatures (20°C for 24 h) and neutral pH (pH 6.7–7.7) and was 4-fold more efficient than the α-galactosidase from green coffee beans for the B-red blood cells conversion into O-red blood cells (Bakunina et al., 1998). Furthermore, a single cold-active α-galactosidase isolated from the Antarctic *Bacillus* sp. LX-1 was reported to be a promising biocatalyst for soybean processing in the food and feed industry (Lee et al., 2012).

Here, we have firstly developed a method for the production of the completely soluble highly active recombinant α-galactosidase of the psychrotrophic marine bacterium *Pseudoalteromonas* sp. KMM 701 (α-*Ps*Gal) in *E. coli*. The use of *E. coli* signal peptide included in the expression plasmid pET40b(+) resulted in overproduction of α-*Ps*Gal. It should be noted that the step of the hybrid protein 6xHis-Dsb-α-*Ps*Gal incubation with enterokinase could be optionally due to the absence of any effect of 32.5 kDa plasmid fusion protein including N-terminal His-tag on the α-*Ps*Gal activity (Figures 1, 2 plasmid scheme and electrophoregram, respectively).

The configuration of the anomeric center of galactose, which was a reaction product of the of 4-NPGP hydrolysed of by α-*Ps*Gal, was monitored by ^1^H NMR spectroscopy. Upon occurrence of signals of anomeric protons at the initial time of reaction one can be appreciate stereochemical configuration of the anomeric center of the formed product. In ^1^H NMR spectra of the initial time of the reaction the resonance of the α-anomeric center of galactose is clearly observed (Figure 3). β-Anomeric as a result of galactose mutarotation appeared only after 10 min from the beginning of hydrolysis reaction of 4-NPGP under the action of α-*Ps*Gal (Figure 4). Mutarotation proceeded until the anomer ratio of 40% α- and 60% β-galactopyranose was established after 60 min, when the reaction was stopped (Figure 4). These observations indicate that the primary product must be α-D-galactopyranose, and the enzyme acts on a mechanism leading to retaining of the anomer configuration of the substrate. Based on these data, we conclude that α-*Ps*Gal catalyzes the hydrolysis of glycosidic bound with double displacement mechanism. This mechanism involves two catalytic residues; one responsible for the protonation of the glycosidic oxygen, and the other for stabilization of a carbocationic intermediate. The glycosyl-enzyme intermediate is decomposed by the transfer of a glycosyl moiety to an acceptor molecule, which is in the case of hydrolysis a molecule of water (Sinnott, 1990). The absence of signals corresponding to the anomeric protons involved in the formation of O-glycosidic bonds indicates the lack of transglycosylation in the conditions of this experiment. It is known that initiation of transglycosylation reaction of galactosidases requires high concentrations of substrates (donors and acceptors) and enzymes, low temperatures and the presence of special solvents (Matsuo et al., 1997; Nieder et al., 2003; Goulas et al., 2009).

Considering the postulation that glycoside hydrolases grouped into the same family shared a common catalytic mechanism (Henrissat and Bairoch, 1993, 1996), all enzymes of GH36 family are retaining glycoside hydrolases. At first the technique of ^1^H NMR experiments was applied for investigation of GH36 enzyme reaction stereochemistry of α-galactosidase from thermopile bacterium *Thermotoga maritima* (Comfort et al., 2007). It was unequivocally shown, that α-galactosidase from *T. maritima* was a retaining glycoside hydrolase. However, this thermophile enzyme has low identity (19.1%) of amino acids sequence with psychrophillic marine *Pseudoalteromonas* sp. KMM 701 (Balabanova et al., 2006). The related catalytic mechanism was shown for GH27 α-galactosidases from *Streptomyces griseoalbus* (Anisha et al., 2011), fungus *Thermomyces lanuginosus* (Puchart et al., 2000), *Phanerochaete chrysosporium* (Brumer et al., 1999), *Trichoderma reesei* (Shabalin et al., 2002). Catalytic mechanism of human α-galactosidase was also studied with ^1^H NMR experiments (Guce et al., 2010).

^1^H NMR experiments were successfully applied for direct detecting of catalytic mechanism of novel inverting GH110 family α-galactosidase from *Bacteroides fragilis* (Liu et al., 2008), as well as for detecting a retaining and inverting properties of GH97 α-galactosidase and α-glucosidase from *Bacteroides thetaiotaomicron* (Gloster et al., 2008).

## Conclusion

For the first time the completely soluble and functional recombinant cold-active α-galactosidase α-*Ps*Gal of marine bacterium was successfully produced in *E. coli* with a high level of yield and specific activity. Based on the results of ^1^H NMR experiments, it was demonstrated that α-*Ps*Gal catalyzes the hydrolysis of substrate with retaining of the anomeric configuration. It is evident that the marine α-galactosidase catalyzes the substrate hydrolyses with double displacement mechanism as all classical glycoside hydrolases of GH36 family of clan-D GH (Comfort et al., 2007; Cantarel et al., 2009).

### Conflict of interest statement

The authors declare that the research was conducted in the absence of any commercial or financial relationships that could be construed as a potential conflict of interest.
